# Supplementary material for: Ag(I) ions working as a hole-transfer mediator in photoelectrocatalytic water oxidation on WO3 film
Source: Nat Commun. 2020 Feb 19;11:967. doi: 10.1038/s41467-020-14775-2 (PMC7031530; doi:10.1038/s41467-020-14775-2)
Supplement: Supplementary file 1 — Supplementary Information [file 41467_2020_14775_MOESM1_ESM.pdf]

Supplementary Information for

Ag(I) Ions Working as a Hole-Transfer Mediator in Photoelectrocatalytic Water Oxidation on WO_3 film

Jeon and Monllor-Satoca *et al.*

Total number of pages: 18

Number of Tables: 2 (1-2)

Number of Figures: 14 (1-14)

Supplementary Table 1. Standard redox potentials ($E°$) of the most relevant silver, oxygen and nitrate mediated charge transfer processes in acidic aqueous solution at pH 0.

**Redox couple**	**Equilibrium**	***E*° (V vs. SHE)**
Ag^+/Ag	$Ag^+ + e^- \rightleftharpoons Ag$	+0.799
Ag_2O/Ag	$Ag_2O + 2H^+ + 2e^- \rightleftharpoons 2Ag + H_2O$	+1.173
AgO/Ag_2O	$2AgO + 2H^+ + 2e^- \rightleftharpoons Ag_2O + H_2O$	+1.398
AgO/Ag^+	$AgO + 2H^+ + e^- \rightleftharpoons Ag^+ + H_2O$	+1.772
Ag^{3+}/Ag^{2+}	$Ag^{3+} + e^- \rightleftharpoons Ag^{2+}$	+1.800
Ag^{3+}/Ag^+	$Ag^{3+} + 2e^- \rightleftharpoons Ag^+$	+1.900
Ag^{2+}/Ag^+	$Ag^{2+} + e^- \rightleftharpoons Ag^+$	+1.980
$O_2/HO_2^\bullet$	$O_2 + H^+ + e^- \rightleftharpoons HO_2^\bullet$	–0.046
O_2/H_2O_2	$O_2 + 2H^+ + 2e^- \rightleftharpoons H_2O_2$	+0.695
O_2/H_2O	$O_2 + 4H^+ + 4e^- \rightleftharpoons 2H_2O$	+1.229
$HO_2^\bullet/H_2O_2$	$HO_2^\bullet + H^+ + e^- \rightleftharpoons H_2O_2$	+1.440
$HO_2^\bullet/H_2O$	$HO_2^\bullet + 3H^+ + 3e^- \rightleftharpoons 2H_2O$	+1.650
$OH^\bullet/H_2O$	$OH^\bullet + H^+ + e^- \rightleftharpoons H_2O$	+2.380
NO_3^-/NO_2	$NO_3^- + 2H^+ + e^- \rightleftharpoons NO_2\,(g) + H_2O$	+0.775
$NO_3^-/\ HNO_2$	$NO_3^- + 3H^+ + 2e^- \rightleftharpoons HNO_2 + H_2O$	+0.940
NO_3^-/NO	$NO_3^- + 4H^+ + 3e^- \rightleftharpoons NO\,(g) + 2H_2O$	+0.957

Supplementary Table 2. Primary particle sizes and particle volumetric ratios of deposited Ag and Ag_2O nanoparticles on WO_3, for Ag(PC)/WO_3 and Ag(PEC)/WO_3 in contact with 0.5 M $NaNO_3$ and 50 mM $AgNO_3$, after 3 h of continuous illumination. Sizes (d) were calculated from the three most intense peaks of XRD patterns and the application of the Scherrer equation ($d = k\lambda/(\beta\cdot\cos\theta)$), where k is an adimensional crystal shape factor (0.891), λ is the Cu Kα emission line wavelength (0.15418 nm), β is the full-width at half maximum (FWHM) of the peak (rad), and θ is the Bragg diffraction angle (º). XRD peaks were fitted to a Lorentzian peak function. As particle sizes lie below 200 nm, β was uncorrected for particle surface strain or instrumental broadening.

**Sample**	**Species**	**2θ (º)**	**β (rad)**	**d (nm)**	**Ag/Ag_2O ratio**
Ag(PC)/WO_3	Ag	38.145	0.00125	116	3.45
	Ag_2O	38.236	0.000868	167	
Ag(PEC)/WO_3	Ag	38.078	0.00131	111	3.15
	Ag_2O	38.180	0.00093	156	

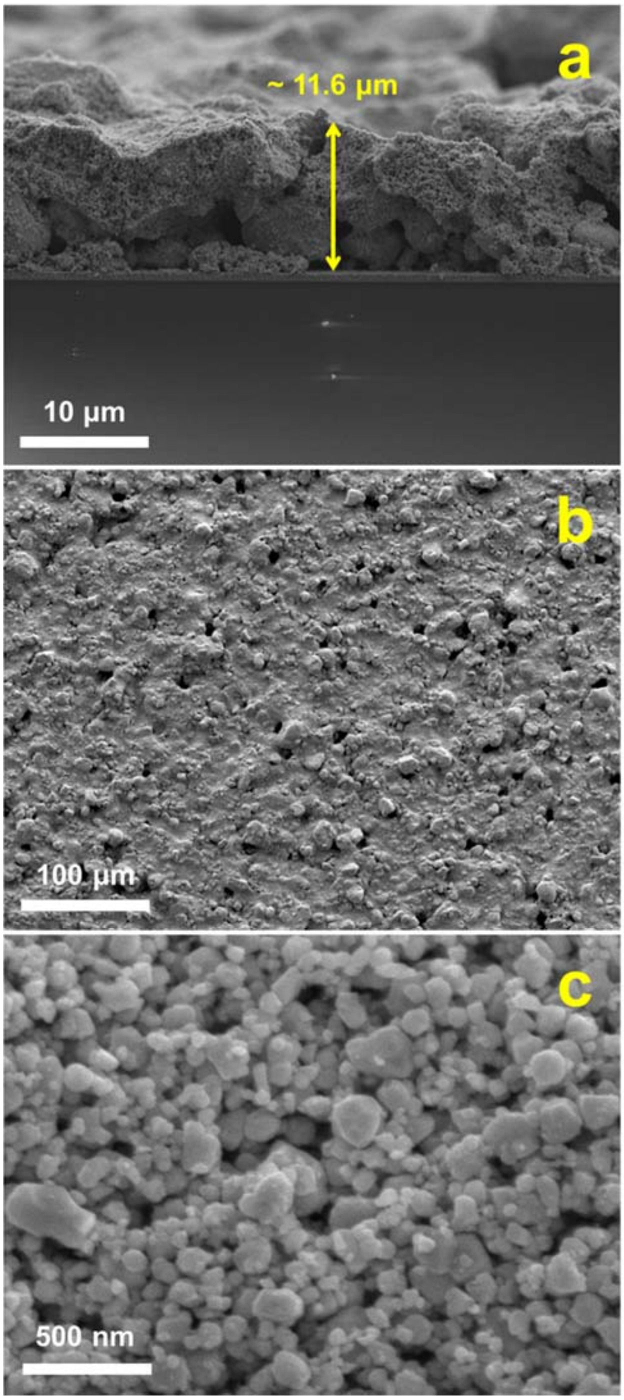

Supplementary Figure 1: SEM of WO_3 film.

SEM micrographs of nanocrystalline WO_3 electrodes, depicting its thickness (a), micrometric nanostructured aggregates (b), and porous character (c).

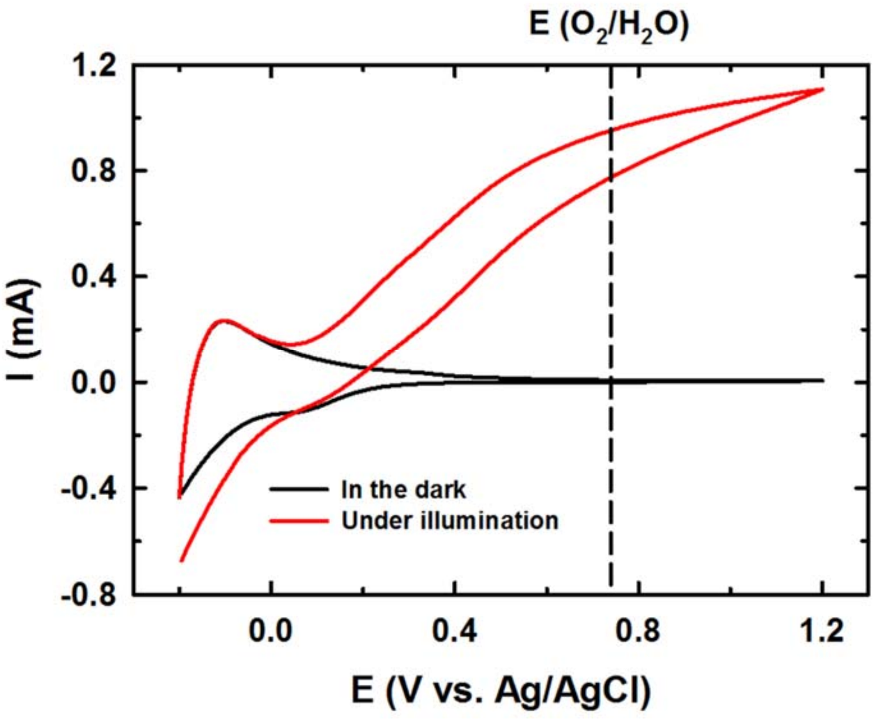

Supplementary Figure 2: CV of WO_3 in dark and under irradiation.

Cyclic voltammograms of nanocrystalline WO_3 electrodes, in the dark (black) and under illumination (red). Scan rate: 20 mV/s. The dashed line represents the thermodynamic value of the O_2/H_2O redox potential at pH 5 (0.74 V vs. Ag/AgCl, equivalent to 1.23 V vs. RHE). Electrolyte: Ar-purged 0.5 M $NaNO_3$, buffered at pH 5.0. Electrode area: 4 cm^2. Irradiation: AM 1.5G (300 mW/cm^2). Source data are provided as a Source Data file.

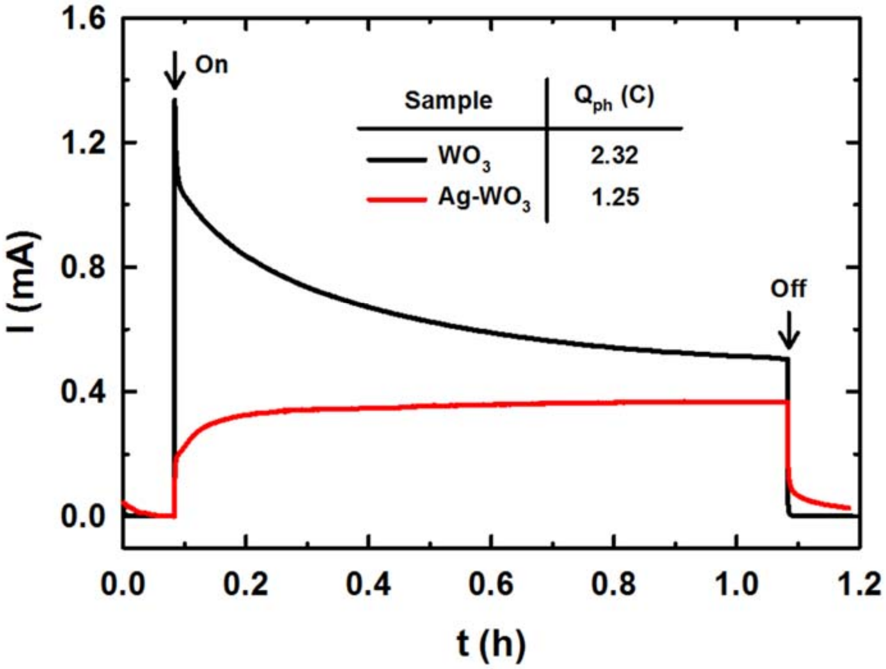

Supplementary Figure 3: Photocurrent-time profiles of WO_3 and Ag-deposited WO_3. Photocurrent transients of an illuminated nanocrystalline WO_3 electrode, before (black) and after (red) silver deposition under open-circuit conditions. Inset: integrated photogenerated charge (Q_{ph}) for each electrode. The silver deposition was performed in the presence of 50 mM $AgNO_3$ during 3 h of continuous irradiation. After irradiation, the electrode was thoroughly rinsed and the electrolyte replaced back to $NaNO_3$. Applied potential: +0.74 V vs. Ag/AgCl (1.23 V vs. RHE). Source data are provided as a Source Data file.

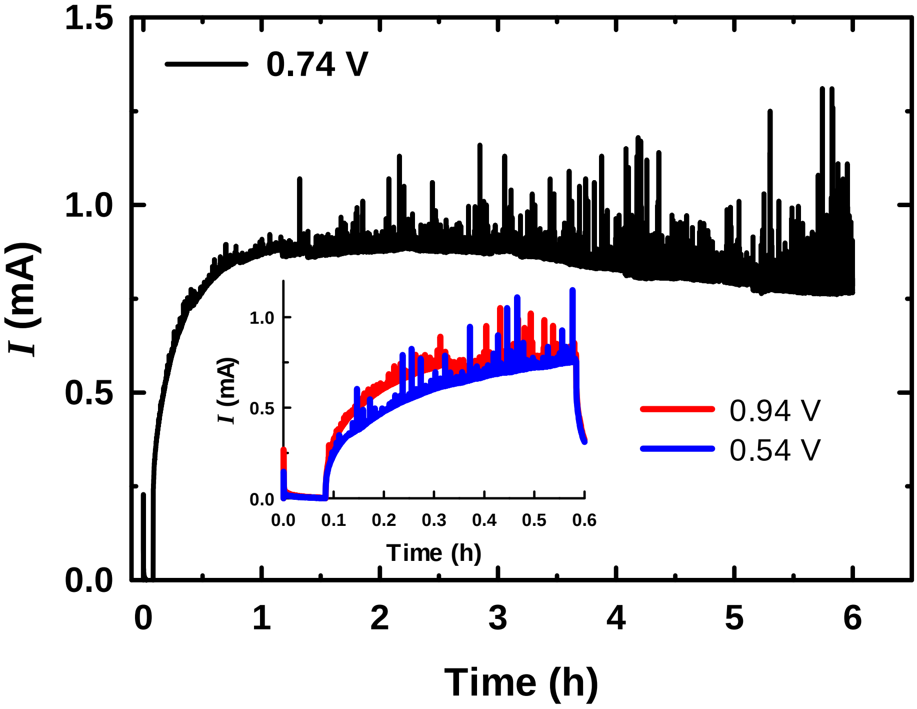

Supplementary Figure 4: Photocurrent-time profiles of WO_3 with Ag(I) at different potentials.

Photocurrent transient of an irradiated WO_3 electrode biased at +0.74 V vs. Ag/AgCl (1.23 V vs. RHE) in the presence of 50 mM $AgNO_3$ and under continuous Ar-purging. The inset shows photocurrent transients of WO_3 biased at +0.940 V vs. Ag/AgCl (red) and +0.540 V vs. Ag/AgCl (blue), which correspond to an overpotential of +0.20 V and –0.20 V with respect to the water oxidation potential (1.23 V vs. RHE), respectively. In all cases, the observed spikes correspond to the release of oxygen bubbles, which suddenly increases the electrolyte-exposed electrode surface area. Other experimental conditions identical to those in **Figure 1**. Source data are provided as a Source Data file.

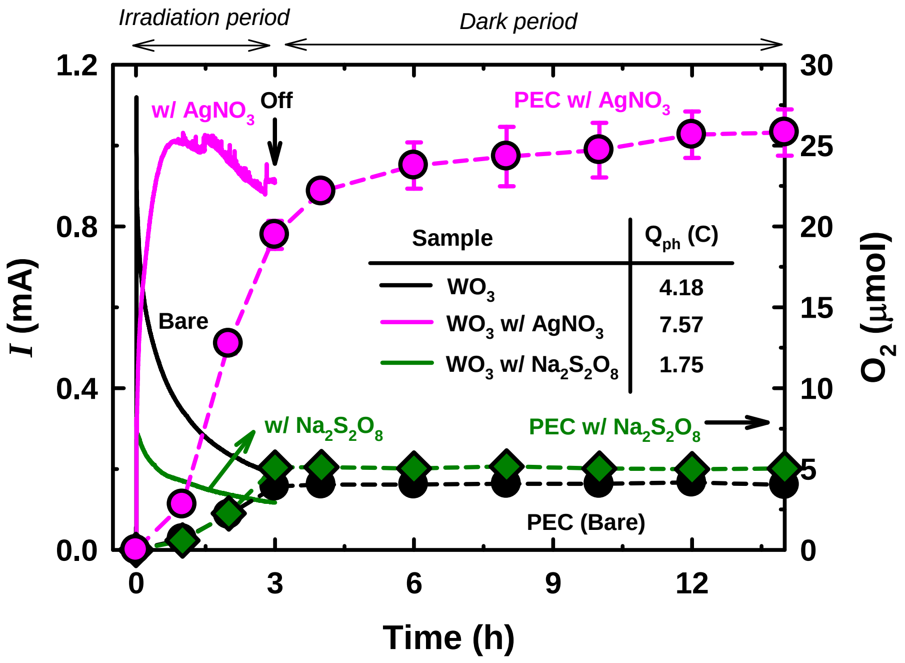

Supplementary Figure 5: Effect of persulfate ($S_2O_8^{2-}$).

Time-profiled photocurrent (left) and concurrent O_2 evolution (right) on a WO_3 electrode biased at +0.74 V vs. Ag/AgCl (i.e., 1.23 V vs. RHE) upon 3 h of continuous irradiation in a sealed cell, with (w/) 50 mM $AgNO_3$ or 50 mM $Na_2S_2O_8$. After turning both potential bias and light off, the amount of O_2 in the headspace was continuously recorded. Other experimental conditions identical to those in **Figure 1**. In the presence of $Na_2S_2O_8$, the photocurrent decreased over 2 times than that in its absence, whereas the amounts of evolved O_2 were similar between the two conditions. This indicates that photogenerated electrons from WO_3 were partially consumed by persulfate ($S_2O_8^{2-}$) acting as an electron acceptor, leading to lower photocurrent generation and higher Faradaic efficiency of O_2 evolution (~100 %). Source data are provided as a Source Data file.

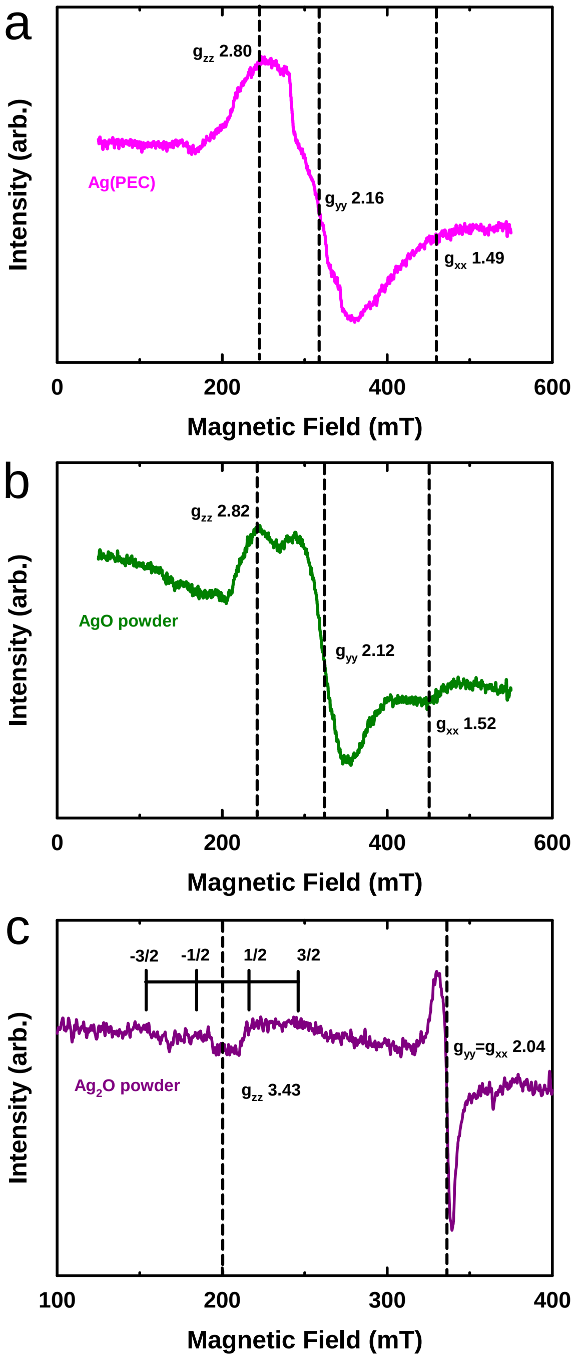

Supplementary Figure 6: EPR spectra of Ag(II) complexes, AgO, and Ag_2O.
Electron paramagnetic resonance (EPR) spectra of (a) brown complexes after PEC reaction at 1.23 V vs. RHE for 3 h, (b) commercial silver(II) powder (AgO), and (c) silver(I) powder (Ag_2O). Source data are provided as a Source Data file.

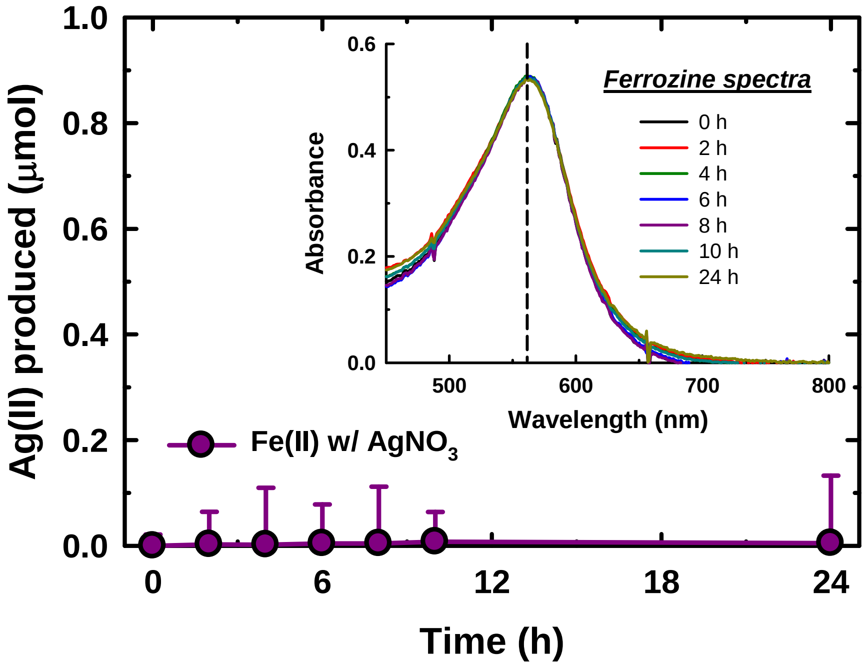

Supplementary Figure 7: Measurement of Ag(II) species by Ferrozine method.

The amounts of Ag(II) produced during the reaction of $Fe^{II}SO_4$ and $Ag^{I}NO_3$. The amounts of Fe(II) were estimated by the Ferrozine method. Inset shows Fe(II)-Ferrozine spectra during the reactions. Source data are provided as a Source Data file.

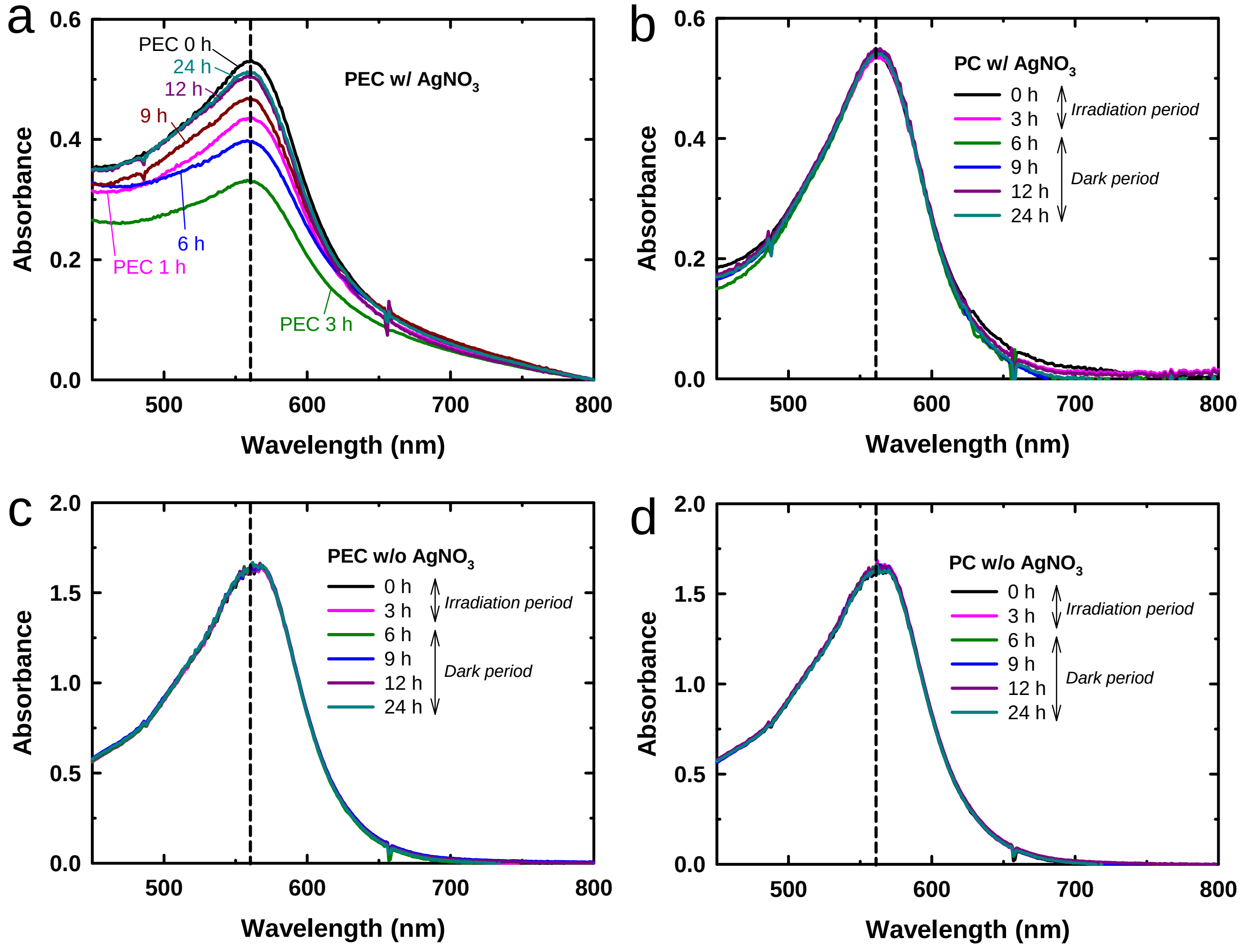

Supplementary Figure 8: Ferrozine spectra for Fig. 4.

Fe(II)-Ferrozine spectra during (a) PEC reaction with $AgNO_3$, (b) PC reaction with $AgNO_3$, (c) PEC reaction without $AgNO_3$, and (d) PC reaction without $AgNO_3$. Source data are provided as a Source Data file.

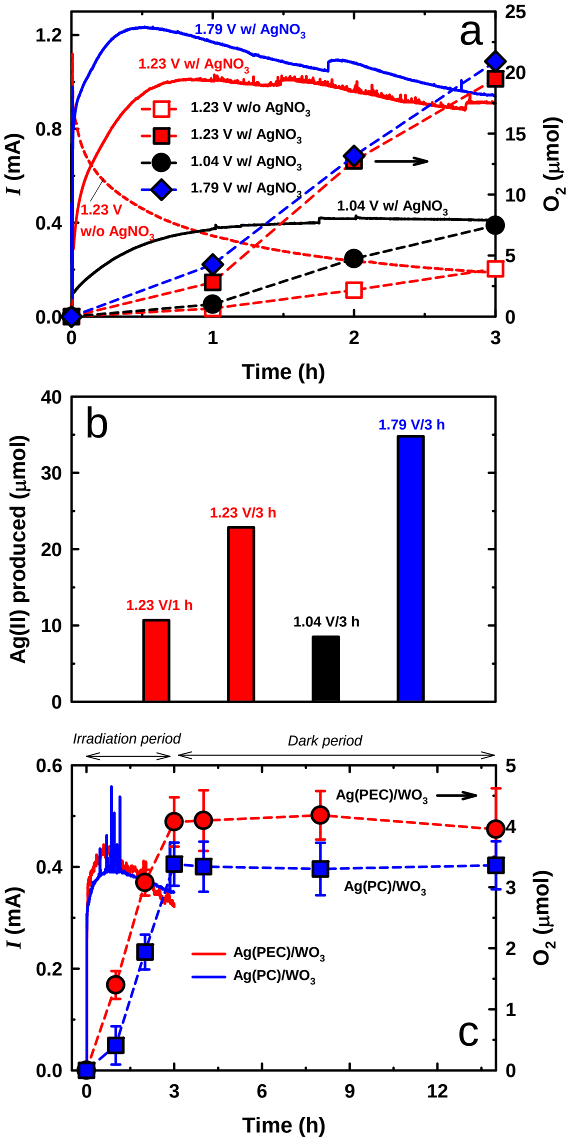

Supplementary Figure 9: PEC performances of WO_3 with Ag(I) at different potentials.

(a) Time-profiled photocurrent (left) and concurrent O_2 evolution (right) on a WO_3 electrode biased at different potentials for 3 h under irradiation in a sealed cell, without (w/o) and with (w/) 50 mM $AgNO_3$. Other experimental conditions identical to those in **Figure 1a**. (b) Ag(II) productions during the PEC periods with $AgNO_3$ for 1 or 3 h at different potentials. (c) Photocurrent profiles and PEC O_2 evolutions using the Ag-loaded WO_3 electrodes (Ag(PEC)/WO_3 and Ag(PC)/WO_3) that were obtained after the PEC reactions with $AgNO_3$ and PC reactions with $AgNO_3$, respectively (see **Figure 1a**). The Ag-loaded WO_3 electrodes were immersed in Ag(I)-free $NaNO_3$ electrolytes (0.5 M) at pH 5 and held at 1.23 V vs. RHE. After irradiation for 3 h, the potential and light turned off and the O_2 evolution was monitored for over 10 h in the dark period. Source data are provided as a Source Data file.

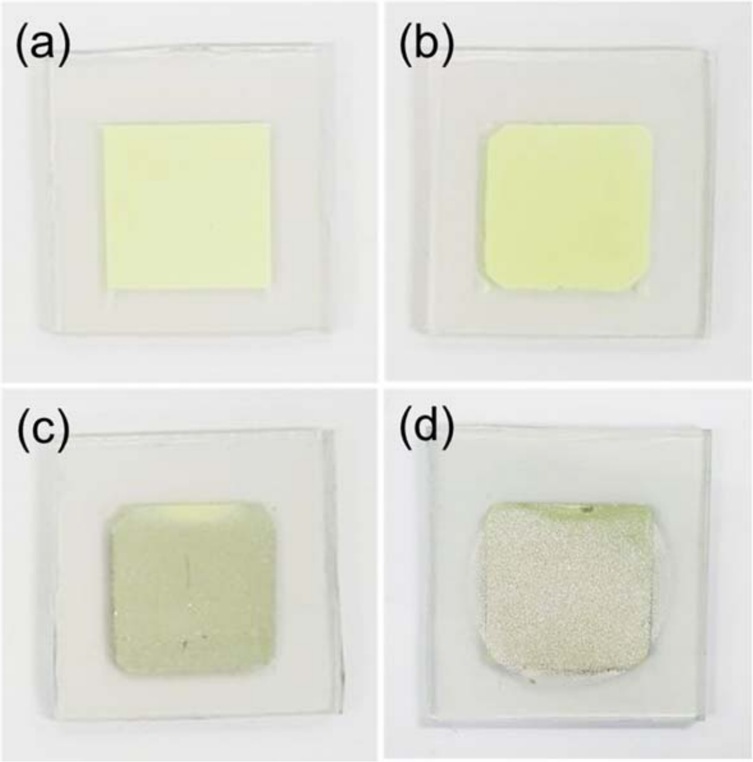

Supplementary Figure 10: Photos of WO_3 and Ag-deposited WO_3 film.

Photos of (a) as-synthesized WO_3 electrode, (b and c) WO_3 electrodes biased at +0.74 V vs. Ag/AgCl under irradiation for 3 h in the absence and presence of $AgNO_3$ (50 mM), respectively, and (d) WO_3 electrode irradiated for 3 h (without bias) in the presence of $AgNO_3$ (50 mM). Electrolyte: Ar-purged 0.5 M $NaNO_3$, buffered at pH 5.0. Electrode area: 4cm^2. Irradiation: AM 1.5G (300 mW/cm^2).

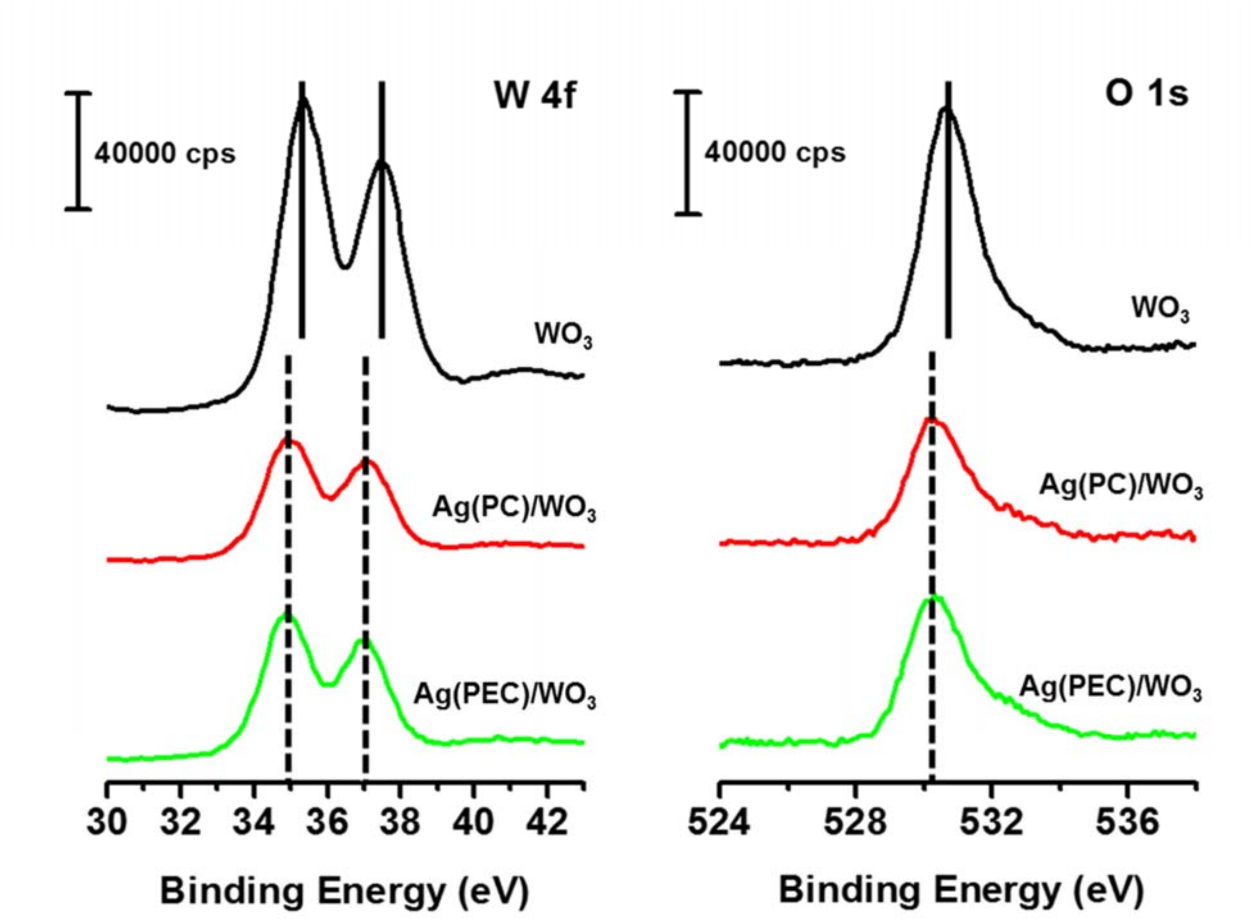

Supplementary Figure 11: XPS spectra of W and O.
XPS W 4f (left) and O 1s (right) spectra of WO_3 (black), Ag(PC)/WO_3 (red) and Ag(PEC)/WO_3 (green) electrodes. XPS peaks: W 4f doublet ($4f_{7/2}$) at 35.0 eV and 36.7 eV; W $5p_{3/2}$ at 41.6 eV; O 1s at 530 eV.

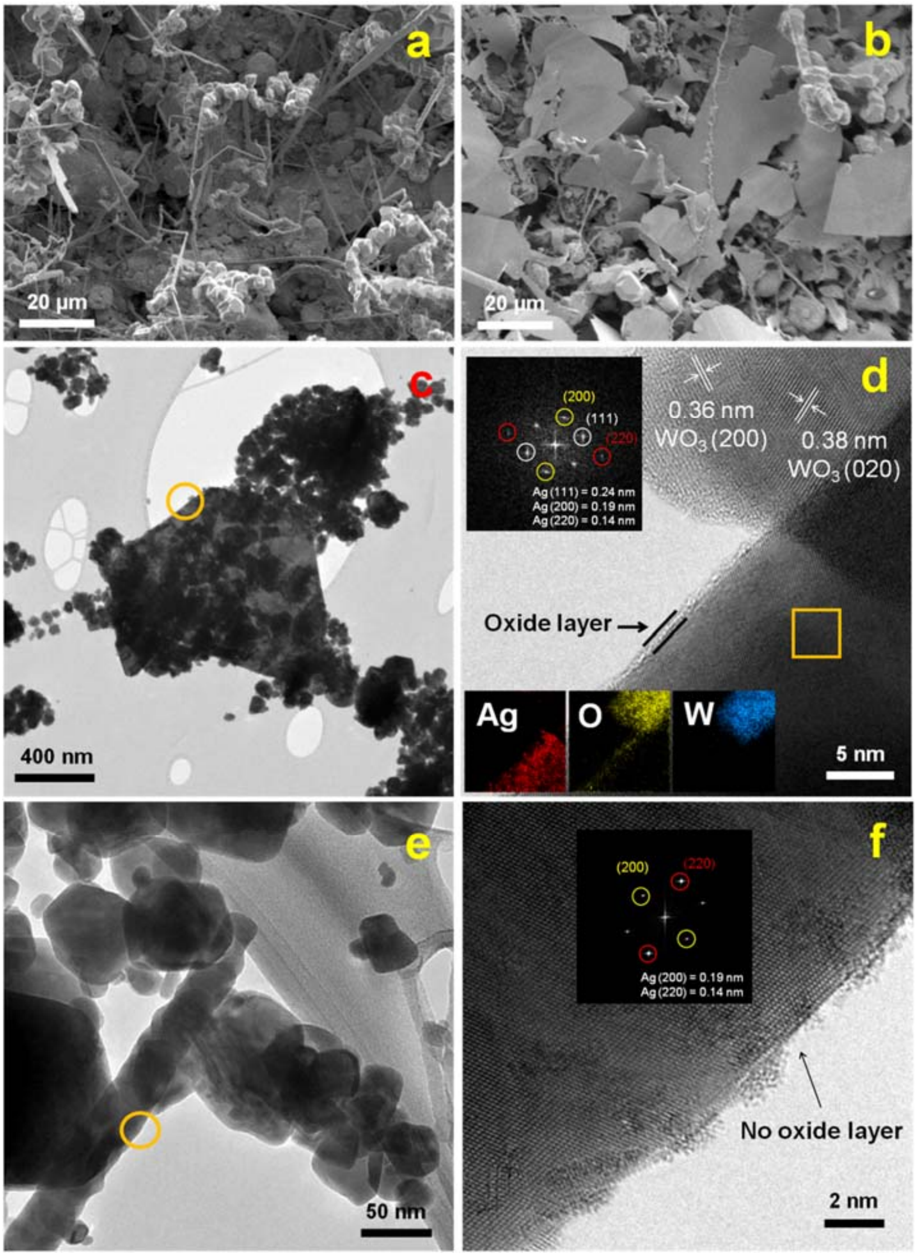

Supplementary Figure 12: SEM and TEM of Ag-deposited WO_3 after PEC reactions.

(a and b) SEM and (c – f) TEM images of Ag-deposited WO_3 particles underwent PEC reactions for 3 h. The samples exhibited heterogeneous mixtures of needle, plate, and particulate configurations. The selected areas (orange squares) in the plates and particles (d and e, respectively) were analyzed for the FFT diffraction patterns. The EELS elemental mappings in d and e confirmed the compositional elements (Ag, O, and W).

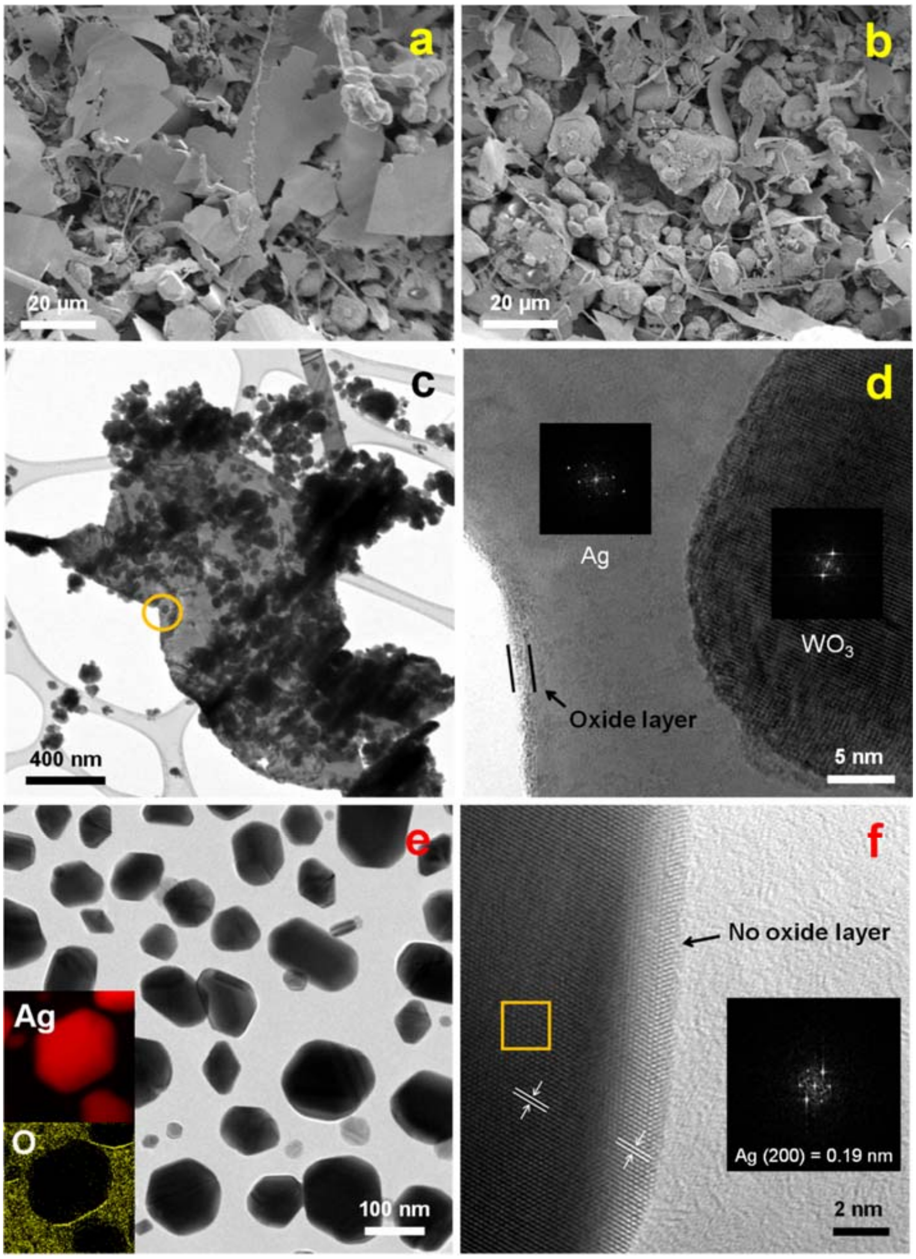

Supplementary Figure 13: SEM and TEM of Ag-deposited WO_3 after PC reactions.

(a and b) SEM and (c – f) TEM images of Ag-deposited WO_3 particles underwent PC reactions for 3 h. The samples exhibited heterogeneous mixtures of needle, plate, and particulate configurations, while the PC sample appeared to have a larger fraction of the plates and more aggregated particles than the PEC one (Supplementary Figure 12). FFT diffraction patterns were analyzed in the areas of the fiber and plates (d and f, respectively). The EELS elemental mappings in c and e confirmed the compositional elements (Ag, O, and W).

The morphology of Ag in the Ag(PEC)/WO_3 showed a heterogeneous mixture of fibers, plate, and particle configurations (Supplementary Figure 12a and b); Ag(PC)/WO_3 sample exhibited the similar morphology of Ag whereas the fraction of plate and the particle size appeared to be rather larger (Supplementary Figure 13a and b). In the PEC sample, the plates of ~1 μm-size were covered with many WO_3 particles with lattice fringe spacing of 0.36 nm (200) and 0.38 nm (020) (Supplementary Figure 12c). The FFT diffraction patterns of the plate interior (bulk) confirmed Ag^0 (111, 200, and 220), whereas the outermost surface of the plates was covered with an amorphous oxide layer (Supplementary Figure 12d). The plates in the PC sample showed the same silver-silver oxide core-shell structure. On the other hand, the hexagonal-shaped particles of max. ~100 nm size were predominantly Ag^0 (200) without any surface oxide layer (Supplementary Figure 13e and f). The absence of the oxide layer was found also in the fibers which appeared to be composed of particles (Supplementary Figure 12e). These SEM and TEM results suggest that Ag(I) should be predominantly reduced to Ag^0 regardless of the presence of the positive potential bias; the Ag particles and fibers (i.e., particle-aggregates) are metallic (Ag^0) whereas the amorphous oxide layer is formed on the surface of the Ag^0 plates presumably by photogenerated holes. Considering that compared to the Ag(PEC)/WO_3 sample, the Ag(PC)/WO_3 sample had a larger fraction of the plates, the Ag^0 produced via the Ag(I) reduction appeared to be partially reoxidized by the holes in the PC reaction. On the other hand, such the hole-mediated oxidation of Ag^0 should be retarded in the PEC reaction because of the enhanced hole transfer under a potential bias followed by the formation of $Ag^{II}NO_3^+$ in solution.

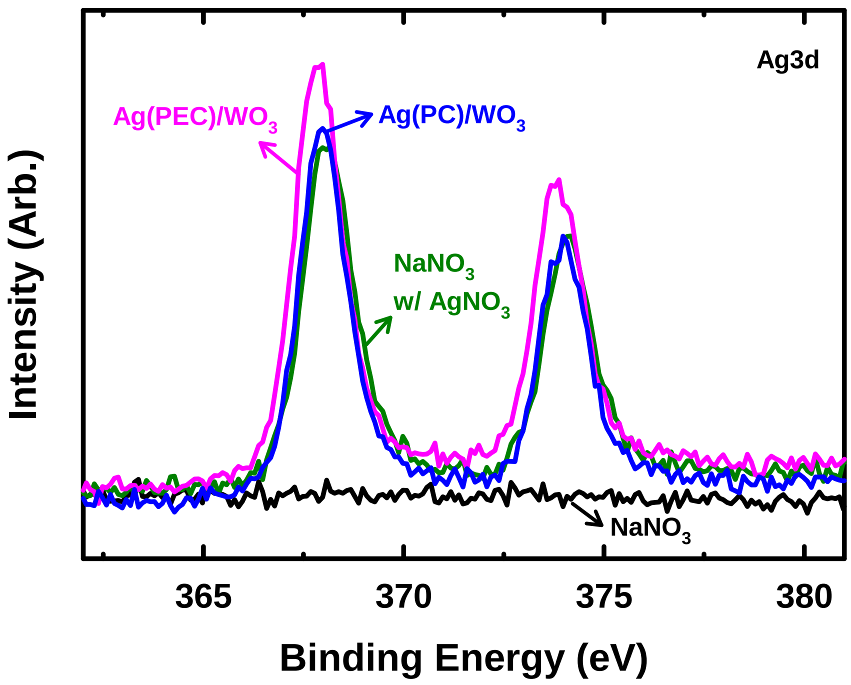

Supplementary Figure 14: XPS spectra of Ag.

Ag 3d XPS spectra of the aliquots collected via adsorption onto silica gel before (i.e., $NaNO_3$ with $AgNO_3$) and after PEC and PC reactions. Source data are provided as a Source Data file.
